# A Recombinant Pseudorabies Virus Expressing Classical Swine Fever Virus (CSFV) E2 Protein Confers Complete Protection Against Lethal CSFV Challenge via Needle-Free Immunization

**DOI:** 10.3390/vaccines14090730

**Published:** 2026-08-24

**Authors:** Ruojia Huang, Qiang Yang, Caoyuan Ma, Xin Song, Tao Wang, Jiaoer Zhang, Chunhao Jiang, Rui Luo, Yongfeng Li, Hua-Ji Qiu, Yuan Sun, Lian-Feng Li, Hongxia Wu

**Affiliations:** 1 State Key Laboratory of Animal Disease Control and Prevention, National High Containment Facilities for Animal Diseases Control and Prevention, Harbin Veterinary Research Institute, Chinese Academy of Agricultural Sciences, Harbin 150069, China; huangruojia2024@163.com (R.H.); 15045381827@163.com (Q.Y.); caoyuanma6@hotmail.com (C.M.); 82101231361@caas.cn (X.S.); wangtao07@caas.cn (T.W.); jiaoerz@126.com (J.Z.); jiangchunhao05@163.com (C.J.); luorui20210423@163.com (R.L.); liyongfeng@caas.cn (Y.L.); qiuhuaji@caas.cn (H.-J.Q.); sunyuan@caas.cn (Y.S.); 2 College of Animal Science and Technology, Heilongjiang Bayi Agricultural University, Daqing 163319, China; 3 Henan Institute of Science and Technology, Xinxiang 453003, China

**Keywords:** classical swine fever virus, recombinant pseudorabies virus, needle-free immunization, protective efficacy

## Abstract

**Background/Objectives:** Classical swine fever (CSF) and pseudorabies (PR), caused by classical swine fever virus (CSFV) and pseudorabies virus (PRV) respectively, are economically significant viral diseases worldwide that severely compromise swine health and constrain international trade. Previously, we generated a recombinant PRV (rPRVTJ-UL44-E2) expressing the CSFV E2 protein that elicited rapid E2-specific antibody responses in rabbits as early as 7 days post-immunization (dpi). However, its immunogenicity and protective efficacy in pigs remain uncharacterized. In this study, we evaluated the protective performance of rPRVTJ-UL44-E2 in pigs via needle-free immunization. **Methods:** Pigs (*n* = 5) received a prime immunization with 10^7^ median tissue culture infective doses (TCID_50_) of rPRVTJ-UL44-E2 via needle-free immunization, followed by a boost immunization with the same dose at 21 dpi, and were then challenged with 10^5^ TCID_50_ of the virulent CSFV Shimen strain (CSFV-SM) at 33 dpi. **Results:** Pigs immunized with rPRVTJ-UL44-E2 developed E2-specific antibodies (blocking rates > cutoff value (40%) at 25 dpi) and gB-specific antibodies that were detectable as early as 7 dpi, with anti-CSFV neutralizing antibody titers comparable to those induced by the C-strain vaccine and anti-PRV neutralizing antibody titers also reaching detectable levels at 28 dpi, whereas no specific antibodies were detected in the DMEM control group. All pigs immunized with rPRVTJ-UL44-E2 and C-strain survived the lethal CSFV challenge with no clinical signs or detectable viremia. In contrast all DMEM control pigs succumbed to infection within 9 days post-challenge (dpc). **Conclusions:** These findings demonstrate that needle-free delivery of rPRVTJ-UL44-E2 confers complete protection against lethal CSFV challenge in pigs.

## 1. Introduction

Classical swine fever (CSF) and pseudorabies (PR), caused by classical swine fever virus (CSFV) and pseudorabies virus (PRV), respectively, remain important viral diseases affecting the global swine industry, particularly in terms of their large impact on pig health and the stringent trade restrictions they impose [1,2,3]. CSF is a highly contagious World Organisation for Animal Health (WOAH)-notifiable disease characterized by high morbidity and mortality, while PR causes annual global economic losses of billions of dollars to the pig industry [4]. Co-infection with the two viruses is frequently reported in endemic regions, often resulting in complicated clinical courses and substantial production losses [4]. Although conventional live-attenuated vaccines have been instrumental in CSF control, the widely used C-strain vaccine lacks differentiation of infected from vaccinated animals (DIVA) compatibility. This deficiency severely complicates serological surveillance and poses a critical bottleneck for CSF eradication programs [5,6].

PRV possesses a large double-stranded DNA genome with multiple non-essential genomic regions, making it an attractive viral vector for bivalent vaccine development [7,8,9]. Deletion of virulence-associated genes such as *gE*, *gI*, and *TK* not only improves the safety profile of recombinant PRV vectors but also enables their use as DIVA-compatible carriers for foreign antigens, allowing for serological differentiation between vaccinated and infected animals. The feasibility of this strategy has been extensively validated. Several recombinant PRV strains expressing the CSFV E2 protein, constructed on either a *gE*/*gI*/*TK*-deleted backbone [10] or a *gE*/*gI*-deleted backbone [11], have been shown to confer complete protection against lethal challenges with both PRV and CSFV in pigs. Beyond conventional expression strategies, recent efforts have further optimized E2 antigen design, including point mutations to enhance full-length E2 expression [12] and employing computer-aided design to generate stabilized E2 variants with improved secretion and thermostability [13], reflecting the ongoing refinement of PRV-vectored CSF vaccines.

In our previous study, we constructed a recombinant PRV (rPRVTJ-UL44-E2) based on a *gE*/*gI*/*TK*-deleted backbone in which the CSFV *E2* gene was fused to the C-terminus of the UL44 membrane protein via a P2A self-cleaving peptide to achieve expression of the E2 protein on the viral envelope [14]. This design aims to preserve the native conformational epitopes of E2, the primary target of anti-CSFV neutralizing antibody (NAb). However, the immunogenicity and protective efficacy of this candidate have not yet been evaluated in pigs.

In recent years, needle-free injection technologies have gained considerable momentum in swine vaccination practices [15]. A recent study demonstrated that, compared with conventional intramuscular injection, needle-free intradermal administration of a recombinant porcine reproductive and respiratory syndrome virus (PRRSV)-vectored CSFV E2 vaccine significantly enhanced humoral immunity and improved average daily weight gain in pigs [16]. In the present study, we therefore employed the same needle-free intradermal immunization approach to deliver rPRVTJ-UL44-E2 and evaluate its protective efficacy against CSFV challenge in pigs.

The present study was designed to comprehensively evaluate the immunogenicity and protective efficacy of rPRVTJ-UL44-E2 in pigs via a needle-free intradermal immunization regimen. We systematically assessed the kinetics of E2-specific antibody responses, the induction of NAb, and the protective efficacy against lethal challenge with a virulent CSFV strain. This study demonstrates, for the first time, that needle-free delivery of rPRVTJ-UL44-E2 confers complete protection against lethal CSFV challenge in pigs. Collectively, this study demonstrates that rPRVTJ-UL44-E2 can be used as a promising DIVA-compatible vaccine candidate and provides critical evidence supporting the feasibility of needle-free immunization for PRV-vectored vaccine applications.

## 2. Materials and Methods

### 2.1. Cell Culture and Virus

The recombinant PRV variant rPRVTJ-UL44-E2 was constructed as described previously [14]. The PRV-TJ strain, initially isolated from a pig herd in Tianjin, China, in 2011 (GenBank accession number: KJ789182.1), and the EGFP-expressing recombinant PRV (rPRVTJ-EGFP) have been characterized previously [17]. The highly virulent CSFV Shimen strain (CSFV-SM; GenBank accession number: AF092448.2), maintained in our laboratory, served as the challenge virus. The C-strain vaccine was obtained commercially from Weike Biotechnology Co., Ltd, Harbin, China.

All four virus strains were propagated in PK-15 cells and stored at −80 °C in our laboratory. PK-15 cells were cultured in Dulbecco’s Modified Eagle’s Medium (DMEM, catalog No. D6429, Sigma-Aldrich, St. Louis, MO, USA) supplemented with 10% heat-inactivated fetal bovine serum (FBS, catalog No. 10091148, Gibco, Carlsbad, CA, USA) and 2% antibiotic-antimycotic solution (catalog No. 15140122, Gibco, Carlsbad, CA, USA), and maintained at 37 °C in a humidified atmosphere containing 5% CO_2_.

### 2.2. Immunization and Challenge Experiment in Pigs

A total of eleven 4-week-old health pigs free of CSFV and PRV antibodies were randomly allocated into three groups: the rPRVTJ-UL44-E2 group (*n* = 5), the C-strain vaccine group (*n* = 3), and the DMEM control group (*n* = 3). Pigs in the rPRVTJ-UL44-E2 group received needle-free immunization with 10^7^ median tissue culture infective doses (TCID_50_) of rPRVTJ-UL44-E2. The C-strain group was immunized with one dose of C-strain vaccine via the intramuscular route. The DMEM group received needle-free injection of DMEM and served as the negative control. A boost immunization was administered at 21 days post-immunization (dpi). Among the parameters, the needle-free injector (catalog No. DERMU-G1-03, DERMU, Jiangsu, China) was set to a pressure value of 75 pounds per square inch (psi) and an injection volume of 0.5 mL. All the pigs were challenged with 10^5^ TCID_50_ of CSFV-SM at 33 dpi. Rectal temperatures and clinical scores were recorded daily. All the surviving pigs were euthanized at 15 days post-challenge (dpc), and tissue samples including inguinal lymph nodes, kidneys, tonsils, and spleen were collected. Clinical scoring was based on five parameters: physical tension, walking, appetite, defecation, and eye conjunctival condition, each graded on a 4-point scale (0 = normal; 1 = mild; 2 = obvious; 3 = severe) [18]. Pigs reaching pre-set humane endpoints (complete anorexia, severe convulsions, and rectal temperature below 37.5 °C indicating imminent death, inability to stand or severe weight loss) were humanely euthanized.

### 2.3. E2-Specific Antibodies Blocking Enzyme-Linked Immunosorbent Assay (ELISA)

CSFV E2-specific antibodies in the serum samples were detected using a commercial blocking ELISA kit (catalog No. 99-43220, IDEXX, Westbrook, ME, USA) according to the manufacturer’s protocol. Results were interpreted based on the antibody-blocking rate. Samples with an antibody-blocking rate ≥ 40% were classified as positive, those with an antibody-blocking rate between 30% and 40% as suspicious, and those with an antibody-blocking rate <30% as negative.

### 2.4. gB-Specific Antibodies Blocking Enzyme-Linked Immunosorbent Assay

PRV gB-specific antibodies in the serum samples were detected using a commercial blocking ELISA kit (catalog No. 99-09732, IDEXX, Westbrook, ME, USA) according to the manufacturer’s protocol. Results were interpreted based on the antibody-blocking rate. Samples with an antibody-blocking rate ≥40% were classified as positive, those with an antibody-blocking rate between 30% and 40% as suspicious, and those with an antibody-blocking rate <30% as negative.

### 2.5. Serum-Virus Neutralization Test

Serum samples were heat-inactivated at 56 °C for 30 min before neutralization assays. Subsequently, the sera were subjected to 2-fold serial dilutions to a final dilution of 1:12,800. Equal volumes (100 µL each) of each diluted serum sample and CSFV-SM (200 TCID_50_) were mixed and incubated at 37 °C for 1 h. The virus-serum mixtures were then transferred to PK-15 cell monolayers in 96-well plates. Following 1 h of adsorption, the inoculum was removed and replaced with DMEM supplemented with 2% FBS. The cells were cultured at 37 °C in a 5% CO_2_ atmosphere for 48 h, and CSFV infection was detected by immunofluorescence assay (IFA) as described previously [14]. The anti-CSFV NAb titers were detected and reported as the log_2_ of the highest serum dilution that conferred 50% neutralization of CSFV infection on the PK-15 cell monolayers. For anti-PRV NAb, the same heat-inactivated serum samples were two-fold serially diluted up to 1:1024. Equal volumes (100 µL each) of each diluted serum and rPRVTJ-EGFP (100 TCID_50_) were combined and incubated at 37 °C for 1 h. The subsequent adsorption, medium replacement and culture procedures were performed as described above, except that cells were incubated for 72 h. PRV infection was identified by direct observation of EGFP fluorescence. The anti-PRV NAb titer was calculated and expressed in the same manner as the anti-CSFV titer.

### 2.6. Reverse Transcription–Quantitative Polymerase Chain Reaction (RT-qPCR)

Total RNA was extracted from anticoagulant-treated samples using a commercial RNA extraction kit (catalog No. DP614, Tiangen, Beijing, China). Subsequently, cDNA was synthesized in a 20 µL reaction system employing avian myeloblastosis virus reverse transcriptase XL (catalog No. KR116-02, Tiangen, Beijing, China). The copy number of the CSFV genome was then quantified by RT-qPCR according to previously established protocols [19].

### 2.7. Histopathological Examination

For histopathological analysis, tissue samples including tonsils and spleens collected from all pigs at 15 dpc were fixed in 4% (*w*/*v*) paraformaldehyde, embedded in paraffin, and sectioned into 5-μm-thick slices. For hematoxylin and eosin (H&E) staining, the paraffin sections were deparaffinized, rehydrated, and stained with H&E according to standard protocols. The stained sections were examined under a light microscope, and representative images were captured for each tissue.

### 2.8. Statistical Analysis

All data were analyzed using GraphPad Prism 8.0.2 and are presented as the mean ± standard deviation (SD). Unpaired two-tailed Student’s *t* test was performed to determine if there are statistically significant differences between the rPRVTJ-UL44-E2-immunized group and the DMEM control group, as well as between the rPRVTJ-UL44-E2-immunized group and the C-strain-immunized group at each indicated time point. Statistical significance was indicated as follows: ns, not significant (*p* > 0.05); * *p* < 0.05; ** *p* < 0.01; *** *p* < 0.001; **** *p* < 0.0001.

## 3. Results

### 3.1. rPRVTJ-UL44-E2 Induced Robust Humoral Immune Responses in Pigs

**Figure 1:**
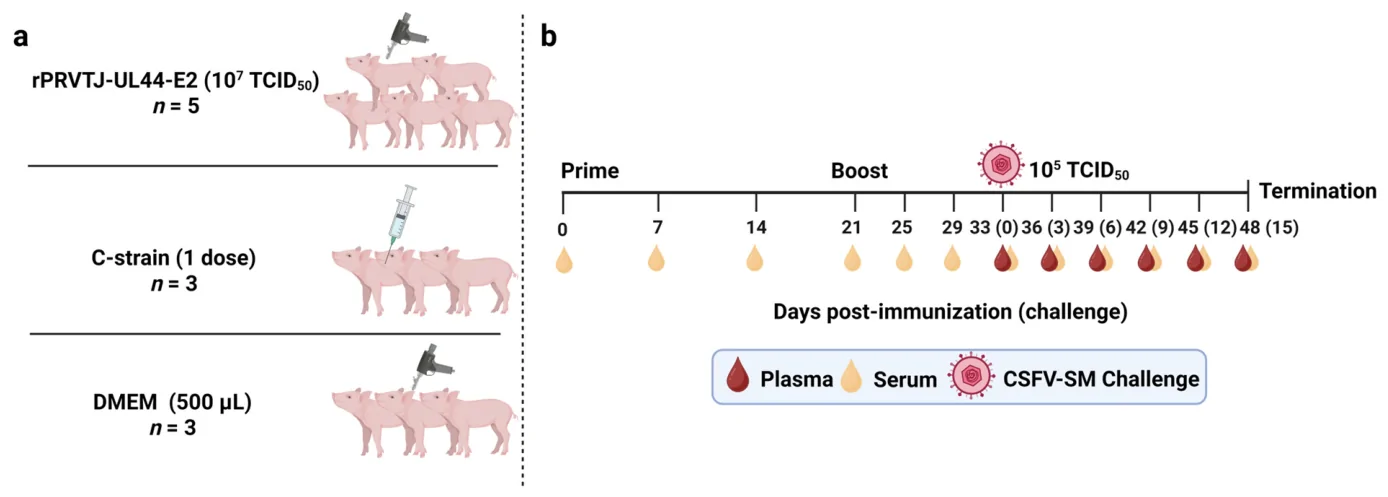
Schematic representation of the immunization procedure in pigs. (**a**) Eleven 4-week-old pigs were randomly assigned to three groups: the rPRVTJ-UL44-E2 group (*n* = 5) received a prime immunization with 10^7^ TCID_50_ of rPRVTJ-UL44-E2 via needle-free intradermal injection; the C-strain group (*n* = 3) was immunized with one standard dose of the C-strain vaccine via the intramuscular route; and the DMEM group (*n* = 3) received DMEM via needle-free injection as a negative control. (**b**) All pigs received a boost immunization with the same dose and route as the prime immunization at 21 dpi. At 33 dpi, all pigs were intramuscularly challenged with 10^5^ TCID_50_ of the virulent CSFV Shimen strain (CSFV-SM). Blood samples were collected at 0, 3, 6, 9, 12, and 15 days post-challenge (dpc) for serological analysis and viral RNA quantification. All surviving pigs were euthanized at 15 dpc for pathological examination.

To evaluate the protective efficacy of rPRVTJ-UL44-E2 in pigs, a total of eleven CSFV-and PRV-negative pigs were randomly allocated into three groups. The immunization doses and experimental groups are shown in Figure 1a, and the immunization schedule and sampling time points and types are shown in Figure 1b.

**Figure 2:**
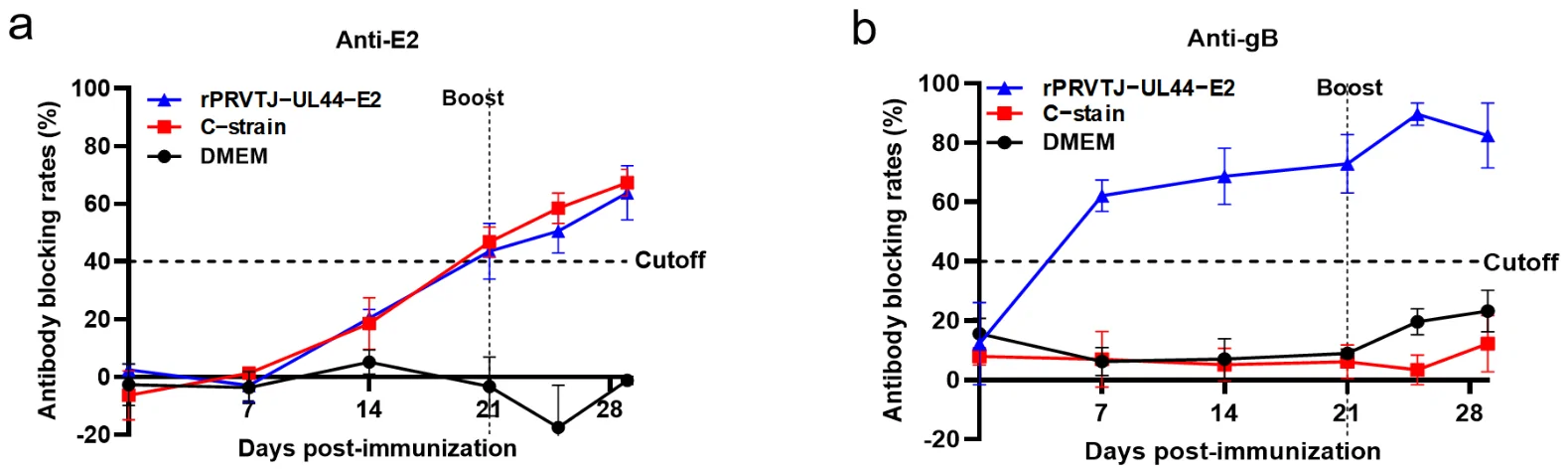
Specific antibodies induced by rPRVTJ-UL44-E2 via needle-free immunization in pigs. Anti-E2 (**a**) and anti-gB (**b**) antibodies induced by rPRVTJ-UL44-E2 in pigs were evaluated by antibody-blocking ELISA.

**Figure 3:**
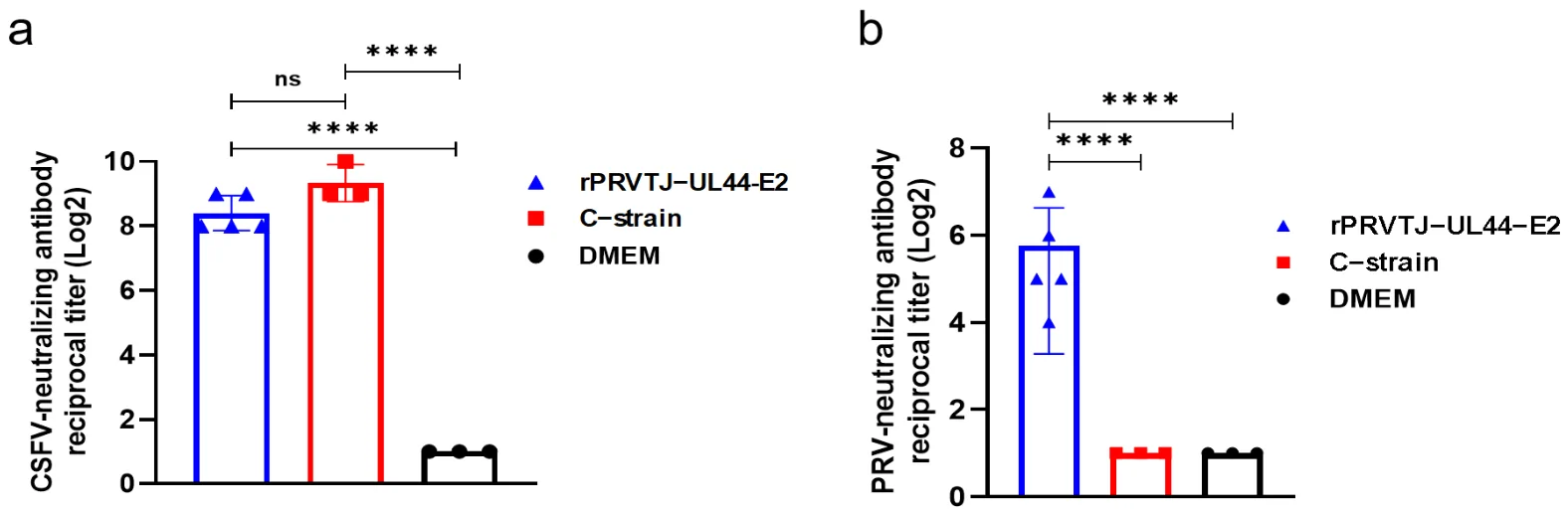
Neutralizing antibody (NAb) titers induced by rPRVTJ-UL44-E2 via needle-free immunization in pigs. Anti-CSFV (**a**) and anti-PRV (**b**) NAb were titrated by serum-virus neutralization test at 29 dpi. Data are presented as mean ± SD. Statistical significance was determined by unpaired two-tailed Student’s *t*-test. **** *p* < 0.0001; ns, not significant (*p* > 0.05).

By 21 dpi, anti-E2 antibody blocking rates exceeded the cutoff value (40%) in all pigs from the C-strain immunized group and in four of five pigs in the rPRVTJ-UL44-E2-vaccinated group. All the pigs of the rPRVTJ-UL44-E2 group achieved blocking rates above 40% by 25 dpi (Figure 2a). Notably, at 28 dpi, CSFV NAb titers in the rPRVTJ-UL44-E2 group were significantly higher than those in the control group, and were comparable to titers induced by the C-strain vaccine (Figure 3a). For PRV, gB-specific antibodies were detected in all immunized pigs as early as 7 dpi, with comparable seroconversion rates across different time points (Figure 2b). PRV NAb titers in the rPRVTJ-UL44-E2 group were also significantly elevated relative to the control group at 28 dpi (Figure 3b) (*p* < 0.0001). Collectively, these data demonstrate that needle-free immunization with rPRVTJ-UL44-E2 elicits E2-specific antibody (blocking rates > cutoff value (40%) in all pigs at 25 dpi) and gB-specific antibody (detected in all pigs as early as 7 dpi) responses, with neutralizing antibody titers against both CSFV and PRV reaching substantial levels by 28 dpi.

### 3.2. Pigs Immunized with rPRVTJ-UL44-E2 via the Needle-Free Route Were Fully Protected Against Lethal CSFV Challenge

**Figure 4:**
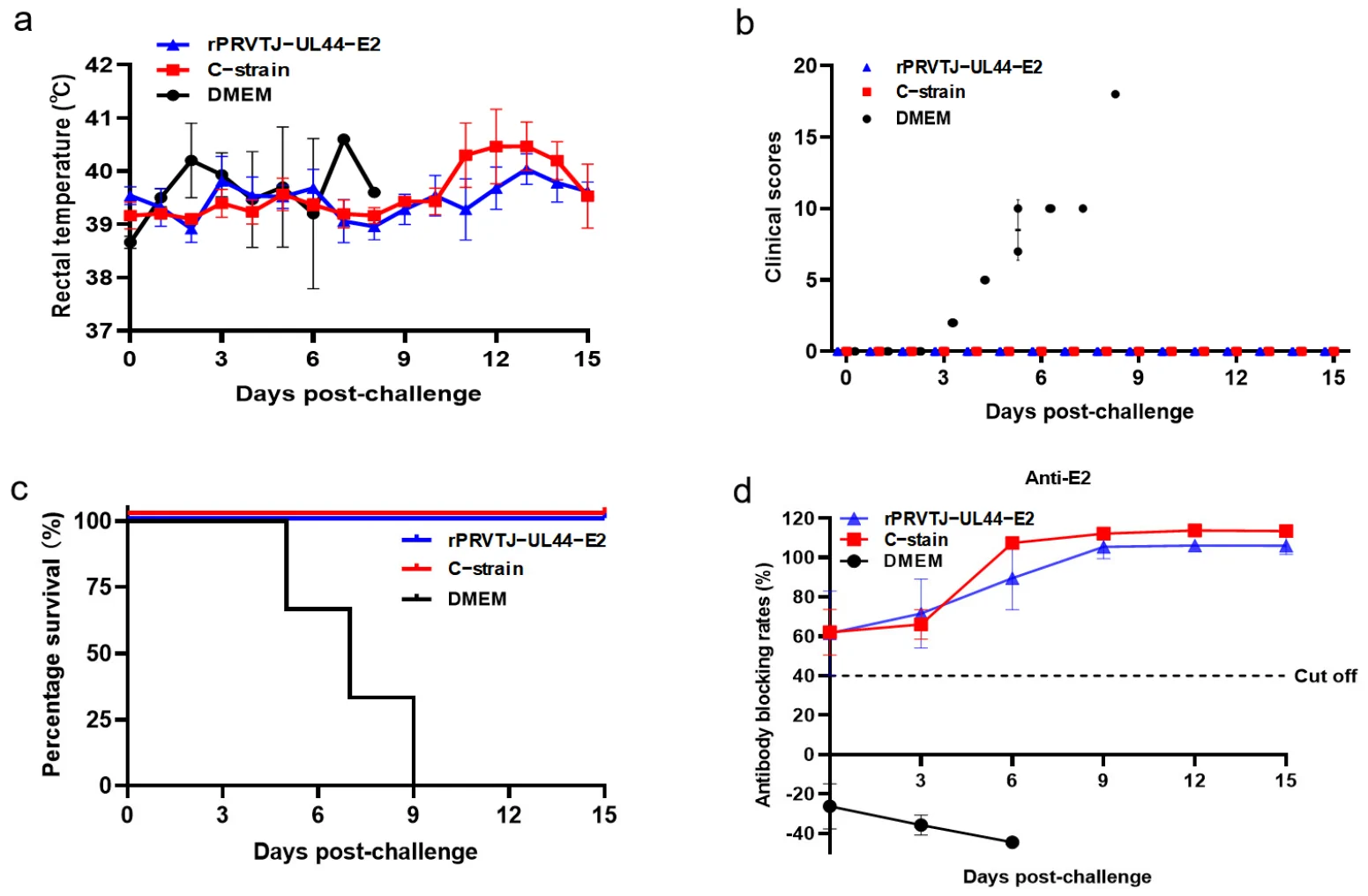
rPRVTJ-UL44-E2 conferred complete protection of pigs from lethal CSFV challenge. (**a**) The rectal temperatures of the pigs following the challenge with 10^5^ TCID_50_ of the CSFV-SM strain. (**b**) Clinical scores based on five clinical signs typical of CSF in the pigs following lethal CSFV-SM challenge. (**c**) Survival rate of the pigs following lethal CSFV-SM challenge. (**d**) Anti-E2 antibodies induced by rPRVTJ-UL44-E2 in pigs were evaluated by antibody-blocking ELISA after lethal CSFV-SM challenge.

Following lethal CSFV-SM challenge, all pigs were monitored daily for rectal temperature and clinical signs for 15 days. By 9 dpc, all the pigs in the DMEM group had met pre-defined humane endpoint criteria and were humanely euthanized. In contrast, all the pigs in the rPRVTJ-UL44-E2 immunization group maintained rectal temperatures below 40.5 °C and showed no clinical signs until the trial endpoint. The C-strain vaccine group remained clinically normal with no elevated body temperature, except for a transient fever that occurred from 11 to 13 dpc (Figure 4a). The pigs in the DMEM group exhibited typical CSF clinical signs, including fever, anorexia, depression, and diarrhea (Figure 4b). All the pigs in both the rPRVTJ-UL44-E2 and C-strain groups survived until the trial endpoint (Figure 4c). To evaluate the anamnestic antibody response upon viral exposure, E2-specific antibody levels were monitored after challenge. As shown in Figure 4d, both the rPRVTJ-UL44-E2 and C-strain groups mounted rapid and strong E2-specific anamnestic responses upon challenge, with blocking rates exceeding 80% by 9 dpc. In contrast, no E2-specific antibodies were detected in the DMEM group at any time point post-challenge. Virological analysis further confirmed the protective efficacy: CSFV genomic RNA was detected in the blood of DMEM controls as early as 3 dpc, whereas no viremia was observed in rPRVTJ-UL44-E2- or C-strain-immunized pigs throughout the observation period (Table 1).

### 3.3. rPRVTJ-UL44-E2 Immunization Prevented Pathological Lesions Following CSFV Challenge

**Figure 5:**
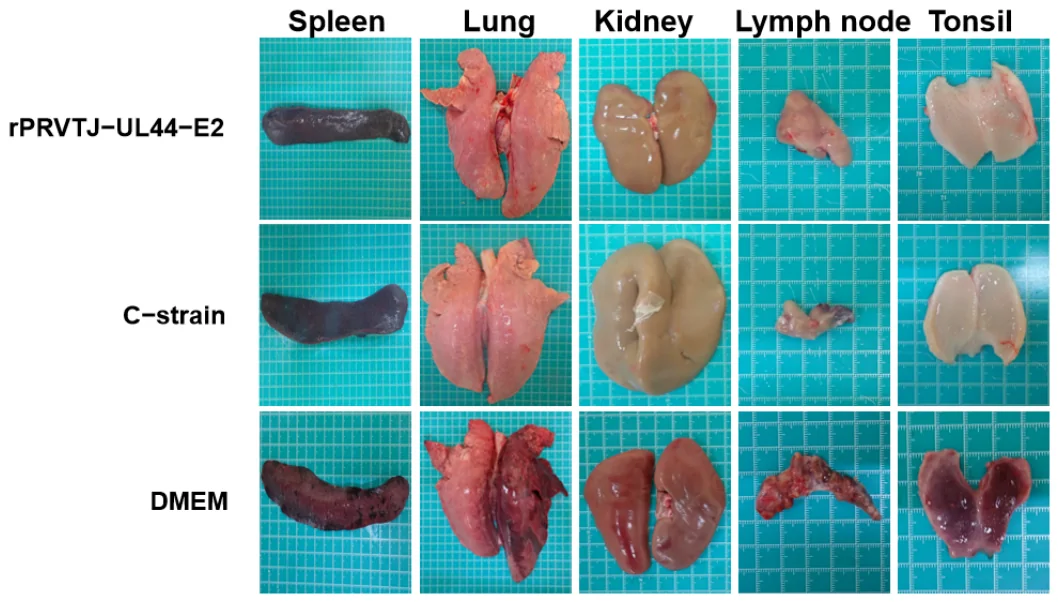
Pathological changes in the immunized pigs following lethal CSFV challenge. Groups of pigs were vaccinated with rPRVTJ-UL44-E2, C-strain, or DMEM. Subsequently, at 33 dpi, all the vaccinated pigs were intramuscularly challenged with 10^5^ TCID_50_ of the CSFV-SM. Surviving pigs were euthanized at 15 dpc. Different tissues, including spleens, lungs, kidneys, lymph nodes, and tonsils, from all the pigs were collected and subjected to general pathological examinations. The green grid background uses a 1-cm^2^ square as the size reference.

**Figure 6:**
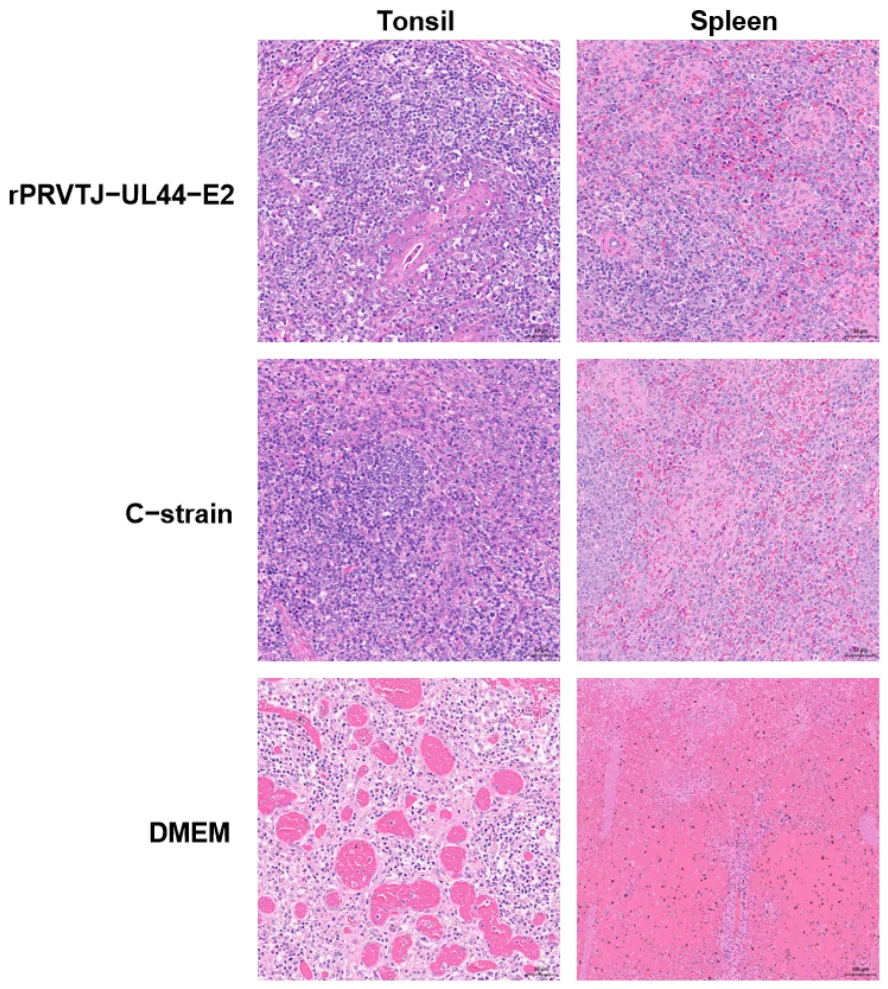
Representative histopathological images of tissue specimens from the tonsils and spleens stained with hematoxylin and eosin staining (H&E). Scale bar = 50 µm (the last one is 100 µm).

Gross pathological examinations revealed no obvious lesions in the rPRVTJ-UL44-E2-immunized group. The C-strain immunized group showed only minor hemorrhages in the lymph nodes, with no other significant pathological alterations. In contrast, the DMEM control group presented with widespread hemorrhages in multiple organs, along with congestion and enlargement of the lymph nodes and tonsils, as well as marginal splenic infarcts (Figure 5). In addition, neither the rPRVTJ-UL44-E2-immunized nor the C-strain-immunized pigs showed any pathological changes. In contrast, pigs in the DMEM control group exhibited marked congestion and loss of tissue architecture in tonsils, as well as significant hemorrhage and structural damage in spleens (Figure 6). Collectively, these findings confirm that needle-free immunization with rPRVTJ-UL44-E2 confers complete protection against lethal CSFV-SM challenge, as evidenced by the absence of clinical signs, pathological lesions, and detectable viremia.

## 4. Discussion

In the present research, we systematically evaluated the immunogenicity and protective efficacy of rPRVTJ-UL44-E2, which expresses the CSFV E2 protein, in weaned pigs via needle-free intradermal immunization. Our results demonstrated that rPRVTJ-UL44-E2 elicited E2-specific antibodies (blocking rates > cutoff value (40%) by 25 dpi in all pigs and NAb titers comparable to C-strain immunization, and conferred complete protection against lethal CSFV-SM challenge.

The rapid and robust anamnestic E2 antibody response observed in rPRVTJ-UL44-E2-immunized pigs following CSFV challenge further supports the establishment of effective B-cell memory. Within 9 dpc, blocking rates exceeded 80%, indicating a strong recall response driven by immunological memory. In the C-strain group, a similar anamnestic pattern was observed. In marked contrast, no E2-specific antibodies were detected in the DMEM control group at any time point after challenge. This absence of seroconversion should not be interpreted as a failure of infection; rather, it reflects the rapid disease progression in these animals. All control pigs succumbed by 9 dpc—a timeframe insufficient for the host to mount a detectable antibody response following primary CSFV exposure, as NAb typically become detectable around 10–14 days post-exposure [6]. Although viral RNA was detected in the blood of control pigs as early as 3 dpc, fatal clinical signs developed before seroconversion could occur. Collectively, these findings indicate that while primary CSFV challenge in pigs may fail to generate detectable antibodies before death, vaccinated pigs are able to mount rapid and robust secondary E2 antibody responses upon viral challenge, further confirming the establishment of effective immune memory.

One point worth further consideration is the relationship between the anamnestic antibody response and viral replication following challenge. Although no viremia was detected in rPRVTJ-UL44-E2-immunized pigs by RT-qPCR throughout the observation period (Table 1), a rapid and marked increase in E2-specific antibody titers was observed between 3 and 6 dpc (Figure 4d). This kinetic pattern is characteristic of a memory B-cell response, which typically requires antigen re-encounter to trigger proliferation and differentiation of memory lymphocytes. It is plausible that the challenge virus underwent a limited period of replication in vaccinated pigs, providing sufficient antigenic stimulation to recall the established immune memory, even though the viral RNA load remained below the detection threshold of the RT-qPCR assay. This phenomenon is not uncommon in vaccinated hosts: low-level viral replication that is insufficient to cause clinical disease or sustain detectable viremia may still effectively boost pre-existing humoral immunity. Additionally, blood sampling alone may not fully capture viral replication in tissues such as tonsils and lymph nodes, which are known sites of early CSFV replication. Thus, while the absence of detectable viremia confirms the absence of systemic dissemination, it does not necessarily exclude the possibility of transient, low-level local viral replication in vaccinated animals.

Needle-free intradermal immunization has emerged as a transformative approach in pig vaccination, offering advantages in animal welfare, biosecurity, and labor safety [15,20,21]. Recent studies have demonstrated that needle-free intradermal delivery of a recombinant PRRSV-vectored CSFV E2 vaccine (rPRRSV-E2) significantly enhanced humoral immunity and improved average daily weight gain in pigs compared to conventional intramuscular injection [16]. In this study, we employed needle-free intradermal immunization for the PRV-vectored CSFV vaccine and observed strong antibody responses (E2 blocking rates > cutoff (40%) by 25 dpi) with complete protection. The vaccine dose used in the present study (10^7^ TCID_50_ per immunization) was selected based on previously established protocols for PRV-vectored vaccines in pigs. Several studies have demonstrated that recombinant PRV strains expressing the CSFV E2 protein confer complete protection at doses ranging from 10^5^ to 10^6^ TCID_50_ [10,11]. Given that this was the first evaluation of rPRVTJ-UL44-E2 in pigs via needle-free intradermal immunization, we opted for a relatively higher dose to ensure sufficient antigen delivery and immune stimulation. The favorable outcomes indicate that the selected dose was appropriate for achieving protective immunity. Nevertheless, we acknowledge that a dose-ranging study has not been systematically performed. Future studies should explore whether lower doses (e.g., 10^6^ or 10^5^ TCID_50_) can achieve comparable protection, which would be relevant for dose-sparing strategies and cost-effectiveness in field applications.

We acknowledge that the number of animals used in this study (*n* = 5 for the rPRVTJ-UL44-E2 group, and *n* = 3 for each of the C-strain and DMEM control groups) is relatively small, which represents a limitation of the present experimental design. However, several considerations justify the sample sizes employed. The sample sizes are consistent with those commonly used in published swine vaccine efficacy studies, including CSFV vaccine studies using *n* = 5 pigs per group, and control groups of n = 3 are also widely accepted [10,22,23]. Furthermore, the sample size was determined in accordance with the ethical principle of Reduction (one of the 3Rs), which advocates for using the minimum number of animals required to obtain statistically meaningful results, particularly in large animal models such as pigs [24]. Despite the limited numbers, the consistency of the observed outcomes across all vaccinated animals—uniform survival, absence of clinical signs and viremia, and strong antibody responses—lends confidence to the reproducibility and biological significance of the findings. Nonetheless, future studies with larger cohorts would be valuable to further validate the protective efficacy of rPRVTJ-UL44-E2.

In conclusion, this study demonstrates, for the first time, that needle-free delivery of rPRVTJ-UL44-E2 elicits protective NAb and confers complete clinical and virological protection against lethal CSFV challenge in pigs. These findings position rPRVTJ-UL44-E2 as a promising DIVA-compatible vaccine candidate and provide critical evidence supporting the feasibility of needle-free immunization for PRV-vectored vaccine applications. Further studies are needed to evaluate its protective efficacy against contemporary PRV variant challenges and to optimize immunization regimens for field use.

## 5. Conclusions

In conclusion, this research provides the first evidence that needle-free delivery of rPRVTJ-UL44-E2 confers complete protection against lethal CSFV challenge in pigs. Further studies are warranted to evaluate its protective efficacy against PRV variant challenges and to optimize the minimum effective dose for field application.

## Figures and Tables

**Table 1:** Quantification of viral RNA in the whole-blood samples from the immunized pigs following lethal CSFV challenge by RT-qPCR.

Group	Pig No.	Genomic Copies (Copies/µL)					
		Days Post-Challenge					
		0	3	6	9	12	15
rPRVTJ-UL44-E2	A1	-	-	-	-	-	-
	A2	-	-	-	-	-	-
	A3	-	-	-	-	-	-
	A4	-	-	-	-	-	-
	A5	-	-	-	-	-	-
C-strain	B1	-	-	-	-	-	-
	B2	-	-	-	-	-	-
	B3	-	-	-	-	-	-
DMEM	C1	-	7.84 × 10^3^	1.00 × 10^5^	/		
	C2	-	1.06 × 10^2^	3.96 × 10^4^	/		
	C3	-	2.11 × 10^3^	/			

-: not detectable; /: died.

## Data Availability

The data presented in this study are available on request from the corresponding authors.
